# Apoptosis Induction of *Agave lechuguilla* Torrey Extract on Human Lung Adenocarcinoma Cells (SK-LU-1)

**DOI:** 10.3390/ijms19123765

**Published:** 2018-11-27

**Authors:** Luis Alberto Anguiano-Sevilla, Eugenia Lugo-Cervantes, Cynthia Ordaz-Pichardo, Jorge Luis Rosas-Trigueros, María Eugenia Jaramillo-Flores

**Affiliations:** 1 Escuela Nacional de Ciencias Biológicas, Instituto Politécnico Nacional IPN; Av. Wilfrido Massieu Esq. Cda. Manuel Stampa S/N Col. Unidad Profesional López Mateos, Alcaldía Gustavo A. Madero, Ciudad de México 07738 Mexico; luis.alberto.anguiano@gmail.com; 2 Centro de Investigación y Asistencia en Tecnología y Diseño del Estado de Jalisco A. C., CIATEJ, Camino arenero 1227, Col. El Bajío del Arenal, Zapopan 45019, Mexico; elugo@ciatej.mx; 3 Escuela Nacional de Medicina y Homeopatía, Instituto Politécnico Nacional IPN, Guillermo Massieu Helguera 239, Col. La Escalera, Alcaldía Gustavo A. Madero, Ciudad de México 07320, Mexico; cordaz@ipn.mx; 4 Escuela Superior de Cómputo, Instituto Politécnico Nacional IPN, Juan de Dios Bátiz y Miguel Othón de Mendizábal S/N Col. Unidad Profesional López Mateos, Alcandía Gustavo A. Madero, Ciudad de México 07738, Mexico; jlrosas@ipn.mx

**Keywords:** *Agave lechuguilla*, apoptosis, cytotoxic activity, molecular docking, mass spectrometry

## Abstract

In this study, an ethanol extract of *Agave lechuguilla* was evaluated against six carcinogenic cell lines (HCT-15, MCF-7, PC-3, U-251, SK-LU-1 and K-562) with an inhibition of 75.7 ± 2.3% against the SK-LU-1 line. Based on the previous result, the extract was hydrolyzed and fractionated, to which the IC_50_ was determined; the cell line was more sensitive to the fractionated extract with an IC_50_ 6.96 ± 0.15 µg/mL. Characterization by mass spectrometry showed the presence of kaempferol, quercetin and a flavonoid dimer formed by afzelechin-4β-8-quercetin, according to the generated fragmentation pattern. The fractionated extract presented cell death by apoptosis with 39.8% at 24 h. Molecular docking was performed with the molecules found to try to describe cell death by apoptosis through death receptors such as FasCD95, TNF-R1, DR4/5 and blocking signaling on the EGFR and K-Ras MAPK/ERK pathway, as well as through the intrinsic pathway activating tBID, which promotes the amplification of the apoptotic signal due to the activation of caspase-3, and consequently caspase-7. In addition to the activation of the IIb complex associated with cell death due to necroptosis.

## 1. Introduction

Cancer is one of the main health problems worldwide; the disease is characterized by abnormal cell growth and invasive behavior [1]. In 2018, lung cancer is the leading cause of death among different types of cancer, with 1,761,007 deaths reported for both sexes in all ages worldwide, representing 18.4% of deaths associated with cancer [2]. The cancer genome is incredibly complex, showing uncontrolled cell growth [3,4], and in particular lung cancer is highly invasive and prevalent disease that arises as a malignant tumor in the cells of the respiratory tract of the epithelium [5].

Histopathologically, it is classified into two groups: Small cell lung cancer (SCLC) and non-small cell lung cancer (NSCLC). Eighty-five percent of lung cancer cases are comprised of NSCLC, and this may include different subtypes of lung cancer such as: Adenocarcinoma, squamous cell carcinoma and large cell carcinoma [6]. The most common lung cancer is adenocarcinoma, which comprises about 40% of all lung cancers [7]. The SK-LU-1 adenocarcinoma cell line presents epithelial morphology and grows in adherent culture, is mutant K-Ras and does not exhibit telomerase activity. The K-Ras gene codes for a G-protein that participates in the EGFR cascade, whose mutations are found on chromosome 12, generating the substitution of an amino acid that determines the constitutive activation of the gene in the form of GTP, and consequently the continuous transmission of the RAF-MAP kinases cascade [7]. Most SCLCs acquire resistance to multiple drugs, whereas NSCLCs tend to be intrinsically resistant to chemotherapy [8]. The EGF receptor is expressed on the cell membrane and patients with lung adenocarcinoma can be classified as EGFR positive or EGFR negative, according to whether or not there is a mutation in the receptor [9]. The causes of lung cancer have been associated with family background with history of the disease [8], use of hormone therapy [9], tobacco smoke in the environment [10], air pollution (increase of 10 µg m^−3^ PM, increase of 10 ppb in SO_2_, increase of 10 ppb in NO_2_) [11], smoke from cooking oil [12], domestic combustion smoke (heating and cooking) [13], patient history (tuberculosis, emphysema, chronic bronchitis, parenchymal infection) [14] and high fruit consumption [15]. During the last decades, flavonoids have gained importance due to their antioxidant capacity and their effects against different types of cancer, reducing cellular viability, migration and invasion, and therefore, their possible therapeutic application [1,16]. *Agavaceae* belongs to the *Agave* genus, which has more than 400 species that grow in semi-arid and arid climates [17,18,19,20]. The *Agave lechuguilla* presents a wide range of secondary metabolites among which there are triterpenes, tannins, volatile coumarins, alkaloids, reducing sugars, steroidal saponins and flavonoids [17,21,22,23,24]. Therefore, the purpose of this work was to determine the cytotoxicity of an ethanolic extract of *Agave lechuguilla*, the hydrolyzate of the extract and the fraction enriched in flavonoids, against lung cells (SK-LU-1), to determine cell death by apoptosis and to elucidate the activation of cell death receptor signaling (extrinsic pathway) and mitochondria-dependent apoptosis (intrinsic pathway) by molecular docking, which also proved to activate molecular targets.

## 2. Results & Discussion

The ethanolic extract of *A. lechuguilla* (EE), has a content of phenols of 23.44 ± 1.47 µg Eq of gallic acid mg extract^−1^, and an antioxidant activity of 1962.99 ± 211.86 μM Eq of Trolox mg extract^−1^, while the hydrolysate (HE) and the enriched extract of *A. lechuguilla* have reduced polyphenols content of 37.45 ± 3.50 and 2.69 ± 0.12 μg Eq gallic acid mg extract^−1^, respectively, Table 1.

By means of UPLC-ESI (+)-MS analysis, the major metabolites were identified in the three different extracts shown in Table 2. The ethanolic extract of *A. lechuguilla* has seven components, five of which are saponins, whose glycones have four to five sugar units between hexoses and pentoses; two flavonoids identified as kaempferol and quercetin according to their fragmentation pattern [17,25,26].

In the hydrolyzed extract, 10 compounds were found, maintaining the signals of kaempferol and quercetin, and due to hydrolysis, the molecular weights 414, 416 and 430 were found, which correspond to different sapogenins found in the ethanolic extract. In addition, two isomers of a flavonoid dimer were found, which presented the same fragmentation pattern, associating the spectra that describe weights corresponding to afzelechin (flavan-3-ol), whose difference in weight between *m*/*z* 579.397 and 304.304 is 275.093 described by [Afzelechin + H] and quercetin (flavonol) with a weight of 302 g/mol reported as [Quercetin + 2H], which conforms to the junction in position (4β-8) since the flavan-3-ols are linked by C4 and the flavanols in C8.

The presence of this biflavonoid identified in the mass spectrum of the extract fraction, could be due to the fact that these compounds are found in very low concentrations that they could not be detected in the ethanolic extract or, during hydrolysis, there were ruptures of polymeric flavonoid units, and this lead to new constituents.

Moreover, five compounds were found in the fractionated extract of *A. lechuguilla*, two of them from the ethanolic extract: kaempferol and quercetin, and the isomers of the dimeric flavonoid.

**Figure 1:**
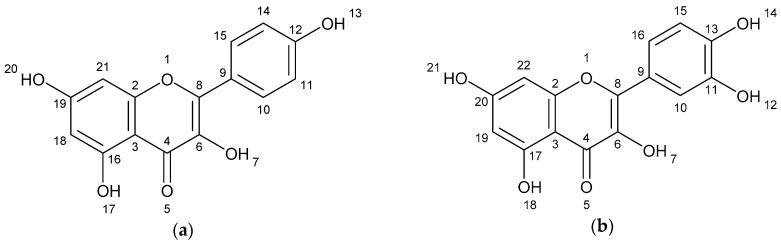

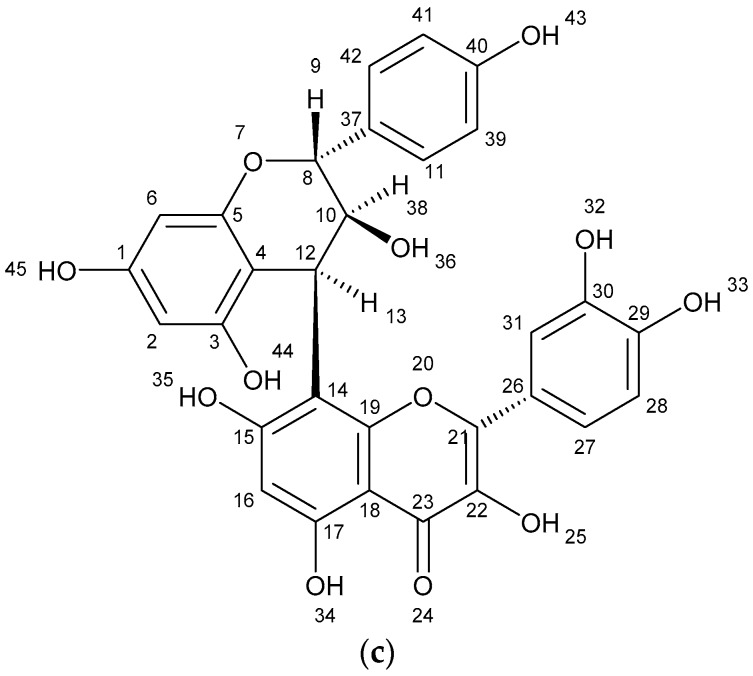
Structure of flavonoids and biflavonoid dimer with numbers associated to *in silico* results. (**a**) Kaempferol 287 g/mol (**b**) Quercetin 302 g/mol, and (**c**) Biflavonoid (afzelequin 4β-8 quercetin), 574 g/mol.

The results of spectometry show the presence of kaempferol (Figure 1a), quercetin (Figure 1b) and the dimer of flavonoid (afzelechin 4β-8 quercetin) (Figure 1c).

Primary screening on six cancer cell lines using ethanolic extract of *A. lechuguilla* at 50 μg/mL (Table 3), showed growth inhibition on the SK-LU-1 cell line in 75.7 ± 2.3% inhibition, followed by the HCT-15 line with 33.4 ± 3.6%, being the most sensitive to the extract, and compared to healthy cells, which were only inhibited in 1.2 ± 0.5%. The most sensitive line against the *A. lechuguilla* extract was SK-LU-1 (lung). Note that the two cell lines (HCT-15 and SK-LU-1) most sensitive to the extract fraction are K-Ras mutants.

**Figure 2:**
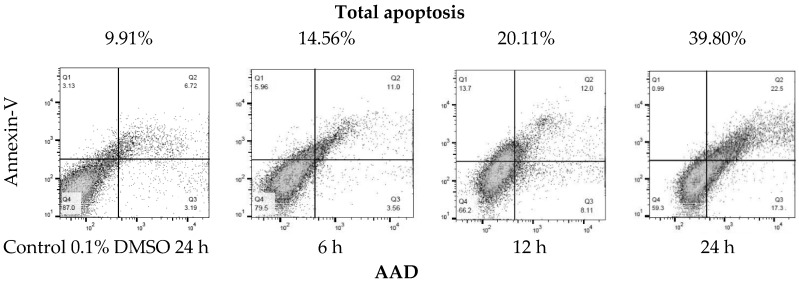
Translocation of phosphatidylserine by flow cytometry (Annexin V-FITC/7-AAD staining). Representative plots of SK-LU-1 cells cultured in the presence fractioned extract of *A. lechuguilla* with an IC_50_ = 6.96 μg/mL, are shown (total apoptosis-time of exposure).

Due to the sensitivity of the SK-LU-1 line to the extract, the IC_50_ of the (a) ethanolic extract (EE), (b) hydrolyzed extract (HE) and the c) extract fraction (EF) of *A. lechuguilla* was determined on the SK-LU-1 cell line. The IC_50_ of the ethanolic extract (EE) was 109.40 ± 0.07 μg/mL, compared to the hydrolyzed extract (HE) of 133.9 ± 0.10 μg/mL, whose value increases due to the presence of free sugars, which probably work as a substrate for the SK-LU-1 cell line, so that the inhibitory power is reduced. Moreover, it was found that for the extract fraction (EF) of *A. lechuguilla* the IC_50_ was 6.96 ± 0.15 μg/mL, the concentration required decreases at the same time by reducing the viability 15.7 times with respect to the ethanolic extract, due to the enrichment of active phenolic compounds, lower concentration of polar compounds and the elimination of free sugars. The results of IC_50_ at 24 and 48 hours of exposure, showed that the most promising treatment is better at higher dose in shorter period. Cytotoxicity data with the ethanolic extract of *A. lechuguilla* found an IC_50_ at 89.0, 128.0 and >150.0 μg/mL of extract against cell lines HeLa, Vero and MCF-7, respectively [27]. Furthermore, for quercetin on lung cells an IC_50_ of 34.15 ± 10.82 μg/mL has been reported [28], a concentration five times higher than the one found by the enriched extract of this study. The extract of the fraction of *A. lechuguilla* turned out to be the one with greater inhibitory effect, therefore, the induction of apoptosis was determined by the translocation assay of phosphatidylserine, demonstrating apoptosis induction. The results show an early apoptosis in the right lower quadrant of Figure 2 (Annexin V^+^/7-AAD^−^) with 3.56% at 6 h and 17.30% at 24 h, and a late apoptosis in the upper right quadrant (Annexin V^+^/7-AAD^+^) that goes from 5.96 to 6 h until reaching a value of 22.5% at 24 h. It was important to include positive and negative controls in this experiment to ensure that the cell population of SK-LU-1 was a true reflection of the events that were taking place after treatment with the fractionated extract of *A. lechuguilla*.

Total apoptosis (programmed cell death) is the sum of early and late apoptosis, as reported in Table 4. At 6 h there is 14.56% of total apoptosis and 20.11% at 12 h, doubling the effect at 24 h with 39.80% total apoptosis. It is highlighted that cell death, due to necrosis, is lower at 24 h with 0.99%, it can be associated that the extract has a controlled death of these cells.

One strategy to improve our comprehension of apoptotic mechanisms, it was in silico studies, that look in space for the most favorable energetic conformation of a protein–ligand complex activating the signaling cascades or inhibiting the receptors as the case may be [29,30]. In T cells type I, cell death signaling by the extrinsic pathway is sufficient to carry out the apoptosis. In this case the SK-LU-1 lung cells are type II, which means that they require an amplification of apoptotic signaling through the extrinsic and intrinsic pathways. The results of the interactions of the ligands and proteins by molecular docking are shown in Table 5; they determine the spontaneity of the reaction due to its negative free energy, hydrogen bonding, polar and hydrophobic interactions. The biflavonoid (afzelechin 4β-8 quercetin) activated the membrane receptor TNF-R1, forming hydrogen bonds with the T89 residue, polar interactions with S81, N92 and E135, as well as non-polar interactions with P90. By the extrinsic pathway, the bond with TNF-R1, activated procaspase-8 to form complex IIa (Caspase-8, FADD and RIP1), and thus, to activate Caspase-8 which in turn activates Caspase 3, and induces apoptosis, or the formation of the IIb complex (Caspase-8, FADD, RIP1, RIP3 and MLKL), thus, activating the necroptosis pathway. Moreover, the activation of DR4 is generated by the formation of hydrogen bonds with T129 and polar interactions with H270, in addition to non-polar interactions with C180 and V152. Experimental data indicate that the residues that activate this receptor (DR4) and its activation, send the signal to acetylate the adapter protein FADD, and to activate caspases 8 and 10, which at the same time activate caspase 3 to trigger apoptosis.

Also by the extrinsic pathway, the binding with the Fas/CD95 receptor acetylates to caspase 8 and 10, this ternary complex is commonly known as DISC composed of Fas, FADD and caspases-8 and -10 [31], which triggers activation of caspase 3, inducing apoptosis. By the intrinsic pathway of the mitochondria-dependent apoptosis, activation by the diflavonoid may be due to the fact that it is a stress promoter for the cell, which culminates in the mitochondrial outer membrane permeabilization (MOMP), allowing the release of proteins from the mitochondrial intermembrane space. This happens in T cells type II because the apoptotic signal needs to be amplified by caspases 3 and 7 [32,33]. In case the biflavonoid crosses the cell membrane, by active transport of K^+^/Na^+^ pumps or even by the calcium–sodium ion exchanger, it can activate the signaling cascade of the binding with the Fas/CD95 receptor by the direct interaction of the biflavonoid with tBID through the R71 and S78 residues; however, BID (BH3 interacting-domain death agonist)is directly activated by caspase 8, generating a tBID, moving to the mitochondria to release cytochrome C (prior stimulation of the Bax–Bak complex, members of the pro-apoptotic Bcl-2 family) [32], to immediately form the caspase-9 Apaf1 complex, which activates caspase-6 and caspase-3 to induce apoptosis. Quercetin interacts with TNF-R1 by hydrogen bonds with residues T89 and T147, by polar interactions with residues N92, N34, T147, P90 and V91, as well as by non-polar interactions, which can generate complexes IIa and IIb; the latter complex mostly has non-polar interactions with RIP1 in residues V75, V76, L78, M92 and L129. Polar interactions with MLKL and residues R210, Q236, E250, K331 and N336 can generate cell death due to necroptosis. At the same time, quercetin activates DR4 by hydrogen bonds with the T129 residue and by E155 by polar interaction, by the extrinsic pathway. By the intrinsic pathway, it could be activated only if it could cross the cell membrane since it only interacts with tBID through hydrogen bonds with R63, R71, and S78, that generate cytochrome C and the activation of caspase-3, and this can activate caspase-7.

Kaempferol is the only molecule that interacts with the Fas/CD95 receptor via a hydrogen bond with the W189 residue, this means that the extrinsic/intrinsic pathways can be equally activated, followed by the RIP complex with caspase-8, -10, activate BID; it becomes tBID through a translocation (that can be activated with interaction by a hydrogen bond with S78), and sends a signal to the BAX complex (polar interactions with D31, N31, S98, T151, S154, L203)-BAK that stimulates cytochrome C to form Apaf-1 with caspase-9 and to induce apoptosis by the activation of caspase-3 (shows hydrogen bonds interactions with H121, Q161, C163, R64, Q161 and A162, and polar interactions R64, E123, Y204 and R207).

In silico studies with ginsenosides (steroids, glycosides and triterpenoid saponins) have shown the activation of Bax with threonine and asparagine residues [34], as in this study. In addition to the DR4 receptors, mostly with polar interactions with T129, E155, R158 and H27, and DR5 R39 and D40. In spite of not activating the TNF-R1 receptor, the action of kaempferol with the complex IIb that generates death by necroptosis was simulated. For this complex, the mechanism where RIP1 and RIP3 present nucleation has been proposed, a model in which the activation of RIP1 and RIP3 kinase and the RIP1/RIP3 amyloid scaffold reinforce each other. The amyloid scaffold can function as a crucial platform to recruit other components, such as MLKL (Mixed Lineage Kinase domain-Like protein), and to trigger the execution mechanisms of necroptosis [35]. RIP1 mostly present non-polar interactions (phosphorylations) with L70, L78, L90 and M92, which add up to necroptosis signaling; MLKL mainly presents polar interactions with residues R210, K230, E250, S335, N336 and E351. From phosphorylated RIP3, the activation of MLKL to phosphorylated MLKL, specifically in the residues of threonine and serine domain in their N-terminus, is necessary, but not sufficient to carry out necroptosis [31,35]. Docetaxel was the ligand as positive control, demonstrating the activation of the signaling cascade in the receptors of the cell membrane of TNF-R1 and DR4/5. Hydrogen bonds were generated in each receptor with T89 for TNF-R1, and R158 and S22 for DR4/5, which activate TRADD acetylases (TNF-R1) that directly stimulate caspase 8 and FADD (DR4/5), to the complex caspases 8 and 10, which at the same time stimulate caspase-3 that triggers cellular apoptosis, all of this via the extrinsic pathway.

Several genetic alterations have been described in NSLC, with EGFR, K-Ras and ALK being the most frequently altered oncogenes, which act as genomic drivers of tumors [36]. EGFR acts as a promoter in cell growth, proliferation and mortality. Kaempferol and the quercetin potently inhibit the intracellular phosphorylation of EGFR, due to its free energy that oscillates between −6.16 to −6.82 Kcal/mol, with polar interactions with D238 and exclusively hydrogen bridges with S262. The complex formed by these flavonoids activates the cytoplasmic domain of the tyrosine kinase receptor. Ras activates Raf kinase, by phosphorylation, and it should be mentioned that Ras mutated to K-Ras in this type of cancer is characterized by the fact that it leads to uncontrolled growth of the cell. However, the results of the molecular docking of K-Ras show a strong interaction of kaempferol, quercetin and the biflavonoid, which together inhibit this proliferation. K-Ras works as a switch (on/off) in signaling. When Ras works normally, it controls cell proliferation, but when it is mutated, control is interrupted and can proliferate continuously. By inhibiting the K-Ras kinase, K-Ras cannot phosphorylate MEK (MEK1 and MEK2), and through molecular docking, it was shown that there is no interaction between MEK and the flavonoids complex, which is favorable to stop the signaling pathway. Molecular docking showed that MAPK activation, despite not being desired, occurs at very high concentrations, 1000 times that of the flavonoids complex (3.08–11.70 × 10^3^ μM) compared to inhibition concentrations against EGFR or K-Ras. That is, the concentration of the flavonoids should be very high to function as promoters in MAPK. By inhibiting this pathway, cell non-proliferation is associated, and since kaempferol, quercetin and biflavonoid activate death receptors by extrinsic and intrinsic pathways associated with apoptosis, cell proliferation decreases.

TGF-β participates both as an activator or suppressor of cell proliferation, suppressing tumors in normal cells, but promoting them in malignant cells [37], while MRP1 acts as a chemoprotector at the cellular interface and in the systemic circulation of multiple tissues [37,38], whose role contributes to drug sensitivity. In both cases by molecular docking, there were no interactions with the flavonoids.

**Figure 3:**
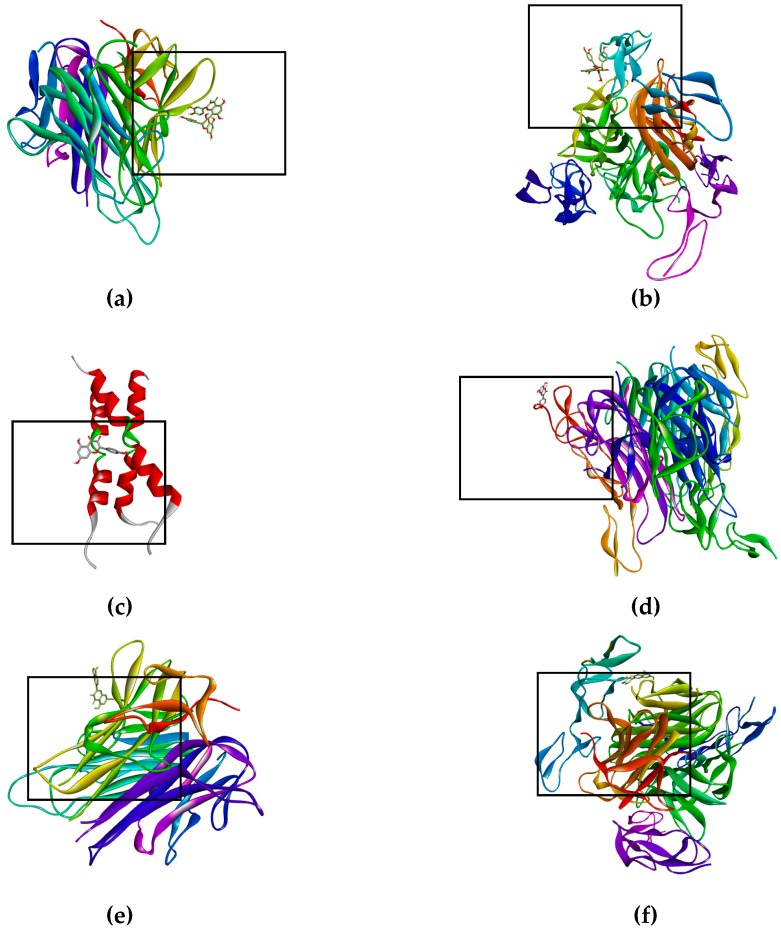

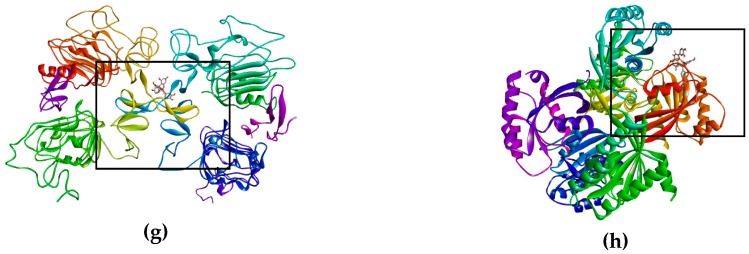
Binds of biflavonoid, kaempferol and quercetin with death receptor. The box indicates the binding region. (**a**) Biflavonoid binds to TNF-R1, (**b**) Biflavonoid binds to DR4, (**c**) Kaempferol binds to Fas/CD95, (**d**) Kaempferol binds to DR5 (**e**) Quercetin binds to TNF-R1, (**f**) Quercetin binds to DR4, (**g**) Biflavonoid binds to EGFR, and (**h**) Biflavonoid binds to K-Ras.

The bioflavonoid (afzelechin 4β-8 quercetin) binds to the receptor TNF-R1 (Figure 3a) and DR4 (Figure 3b), and quercetin binds to TNF-R1 (Figure 3e) and DR4 (Figure 3f), activating death cell signaling by apoptosis; previously explained. Biflavonoid and the quercetin, activate the same death receptors, present the same type of interactions by hydrogen bonds with T89, polar interactions with N92 and non-polar interactions with P90, for TNF-R1; while for the DR4 receptor they only generate similar hydrogen bonds with T129. This may be due to the fact that the flavonoid contains a unit of quercetin in its molecule, part of these interactions are the same as those presented for quercetin in the form of a monomer. The interaction shown by kaempferol, quercetin and the dimer of flavonoid with tBID, demonstrates the ability to amplify the apoptotic signaling, while kaempferol binds to DR5 (Figure 3d) and Fas/CD95 (Figure 3c) which has the peculiarity of activating the intrinsic pathway, and the kaempferol interaction with caspase-3 that helps the same amplification of the apoptosis.

The proteins of the Bcl-2 family can be classified into anti-apoptotic and pro-apoptotic, since there is a fine balance between both by homeostasis, if there is an overexpression of the anti-apoptotic proteins of the BCL-2 family, it prevents the release of cytochrome C from the mitochondria [34], given the results of simulation, no interactions with Bcl-2 were found; however, kaemperol with the Bax protein, instead of Bak, stimulates the apoptotic signaling cascade. Several studies have shown the activations against specific molecular targets, however, they have not studied the activations of other proteins that amplify the signaling of apoptosis by molecular modeling. In contrast, this study showed that the extract obtained can function as an activator of death receptors, as well as acting as an activator of molecular targets that amplify apoptosis signaling, such as caspase-8 and Bcl-2, in NSCLC [39,40].

On the other hand, there are receptors where inhibition is sought, as was the case of EGFR (Figure 3g) and K-Ras (Figure 3h), where having high affinity of the ligand for the active site of the receptor (EFGR) and of the protein (K-Ras), thus blocking the signaling cascade that promotes the proliferation of lung cancer cells SK-LU-1.

In addition to the participation of flavonoids in the aforementioned signaling pathways, these also participate in improving the immune response, which may contribute to ameliorate the health status. It is important to consider that even though chemotherapy continues to be the best treatment for cancer, resistance to drugs results in the reduction of intracellular levels of drugs at concentrations lower than therapeutic levels, resulting in poor chemotherapy. This resistance to drugs is increasing, so part of the solution is the search for compounds that selectively kill multidrug resistant cells (MDR), but not the parental cells from which they are derived (non-resistant), a phenomenon called collateral sensitivity (CS). CS is a type of synthetic lethality, wherein the genetic alterations accrued while developing resistance to one agent is accompanied by the development of hypersensitivity towards a second agent [41]. One of the four postulated mechanisms for this collateral sensitivity is the modulation of reactive oxygen species, where antioxidant compounds, such as flavonoids, become relevant. Given the accumulated evidence of the anticancer effects of flavonoids, coupled with little or no damage to normal cells, such as natural antioxidant quercetin whose LD50 is 160 mg/kg body weight, placing it as a potential therapeutic agent for be used in complementary therapy, however, there is also contradictory evidence, since on the one hand it is recognized as an anticancer agent, antiproliferative, chemopreventive, anti-inflammatory, even at the level of clinical trials, but on the other hand it is reported that 2-year studies quercetin showed carcinogenic activity in the kidney of the male rat, at a dose of 40–1900 mg/Kg/day. It is also well known that phenolic compounds at high doses can behave as pro-oxidants and present undesirable effects [42,43].

## 3. Materials and Methods

### 3.1. Plant Material

The *A. lechuguilla* was collected in San Bartolo (23°09′34.0′′ N 100°34′10.7′′ W) municipality of Villa De Guadalupe, San Luis Potosí State, Mexico in August 2017. The ripe leaves (25–50 cm) were air-dried (40 °C) and powdered (100 mesh).

#### 3.1.1. Ethanolic Extract from *A. lechuguilla*

The dried powder of *A. lechuguilla* was extracted by sonication for 90 min with 96% ethanol (1:10 *w*/*v*). All the solvent was evaporated to dryness under reduced pressure (40 °C) (Ethanolic extract).

#### 3.1.2. Hydrolysis Extract and Its Fractionated Hydrolyzed Extract of *A. lechuguilla*

Five milliliters of 50% aqueous methanol containing 1.2 M HCl and 0.04% (*w*/*v*) ascorbic acid as antioxidant were added to 50 mg of ethanolic extract dried sample. The hydrolysis was performed at 80 °C under reflux for 2 h and the extract was allowed to cool; the extract was subsequently diluted to 10 mL with methanol [44]. For subsequent analyzes 7 mL were evaporated to dryness under reduced pressure (40 °C) (Hydrolyzed extract of *lechuguilla*).To enrich the extract for phenolic compounds, 3 mL of hydrolysis extract of *A. lechuguilla* were eluted through a C18 cartridge (Sep-Pak classic, Part No: WAT051910, Water Co.), previous solvation with 3 mL H_2_O (3×); then the cartridge was eluted again with 3 mL H_2_O (3×) and the final 3 mL EtOH (3×) (Fraction extract of *A. lechuguilla*). All the solvent was evaporated to dryness under reduced pressure (40 °C).

### 3.2. Polyphenol and Flavonoid Total Quantification

The content of total phenolic compounds was determined by the Folin–Ciocalteau method [45] and the total of flavonoids compounds was quantified by the aluminum trichloride method [46].

### 3.3. Antioxidant Capacity Assays

The antioxidant capacity was determined by TEAC [47] and ORAC [48].

### 3.4. Mass Spectrometry

The samples were filtered (0.22 μm) with an injection volume of 3 μL, by using a BEH C18 1.7 μm column (Acquity UPLC, Waters) at 30 °C, with a flow of 0.3 mL/min with the following program: Solvent A (ACN) and solvent B (Water pH 2.5 TFA) 15% A 0 min, 35% A at 6.97 min, 35% at 15 min. The sample separated by the column was nebulized by ESI-Q-ToF (Xevo G2-XS QTof, Waters, Milford, MA, USA) and the ions that were collected in the range of 100 to 1300 Da, in positive (+) mode with the MassLynx software (v4.1, Waters, Milford, MA, USA) and the data were analyzed in the MestReNova software (v12.0.20910, Mestrelab Research S.L., http://mestrelab.com/)

### 3.5. Screening Cells Viability Assay by Sulforhodamine B

The cell viability determination of the ethanolic extract of *A. lechuguilla* (50.0 µg/mL) against six different cell lines was carried out: K-562 (chronic myeloblastic leukemia), HCT-15 (colon), MCF-7 (breast), PC-3 (prostate), U-251 (central nervous system) and SK-LU-1 (lung), with doubling times of 19.0 h, 18.1 h, 25.6 h, 28.7 h, 25.4 h and 25.4 h, based on this, the cell density was calculated 5000, 10,000, 5000, 7500, 7500 and 7500 cells/well, respectively; additionally, the cell line Cos-7 (monkey kidney) was used as control. The cell lines were provided by the Biological Testing Laboratory of the Chemistry Institute (Laboratorio de Pruebas Biológicas del Instituto de Química, UNAM, Mexico City, Mexico). The cells grew in RPMI-1640 culture medium supplemented with 10% FBS and with a mixture of 10.0% antibiotics-antimycotics and 2 mM glutamine. With the exception of line K-562, the remaining cell lines adhere to the culture bottles, to harvest them 1 mL Trypsin-EDTA (0.05%) is added, then the cells are detached from the plastic substrate by adding between 5–10 mL of culture medium to inactivate the trypsin. The cell suspension is centrifuged for 3 min (2000 RPM). Once the cellular package is formed, the culture medium is added to suspend them. Thirty microliters of inoculum are taken and mixed with 30 μL of trypan blue. The inoculum corresponds to 100 μL/well at the density described above. Each microplate was inoculated with two cell lines in triplicate, and incubated for 24 h at 37 °C, in an atmosphere of 5% CO_2_ and 95% relative humidity. The ethanolic extract of the *A. lechuguilla* was dissolved in DMSO (Sigma-Aldrich, St Louis, MO, USA), to form a stock solution stored at −20 °C. The stock solution was dissolved in culture medium to obtain 0.1% DMSO, then it was sterilized by filtration prior treatment and 100 μL were added, with a final volume of 200 μL/well. The microplate is incubated for 48 h under the same conditions previously mentioned. At the end of the incubation of the extract, the cell lines were fixed in situ by adding 50 μL of cold TCA 50.0%, incubated at 4 °C for 60 min, the supernatant was discarded, and the plates were washed five times with deionized water and dried at room temperature. The staining of the fixed cells was performed with a 0.4% SRB solution and incubated for 30 min at room temperature. The unbonded FBS was washed three times with 1.0% acetic acid and dried at room temperature. 100 μL of Tris buffer were added to the stained microplates and shaken for 10 min. The optical density was measured at λ-515 nm to the stained microplates [49]. Optical densities were averaged for the wells treated with the compound (ODt), the wells treated with DMSO (ODc) and the control wells, i.e. those that do not have cells, but have a compound (ODb). The IC was calculated with the following formula [50]:

% IC = 100 − (ODt − ODb)/(ODc − ODb) × 10(1)

### 3.6. Viability Assay by 3-(4,5-DiMethyl-2-Thiazolyl)-2,5-Diphenyl-2H-Tetrazolium Bromide

The cell viability through MTT formazan (Sigma-Aldrich, MO, USA) by the mitochondrial succinate dehydrogenase is present only in living cells. The absorbance value of formazan is directly proportional to the number of viable cells [51]. The fraction of *A. lechuguilla* extract was dissolved in DMSO (Sigma-Aldrich, MO, USA), to form a stock solution that was stored at −20 °C. The stock solution was dissolved in RPMI-1640 culture medium supplemented with 10% FBS and 1% antibiotic-antimycotic to obtain 0.1% DMSO, then it was sterilized by filtration prior treatment [52], they were exposed with the extracts at 24 and 48 h, with extract concentrations of 0.3 up to 300 μg/mL, DMSO was used as negative control (0.1%), and as positive control (5.0 μg/mL) (Paclitaxel from *Taxus brevifolia*, Sigma, Cat. No. T7402). The 96-well microplates were incubated with 8000 cells/well at 37 °C, 5% CO_2_ and 95% relative humidity for 24 and 48 h. At the end of the treatment, they were removed and washed with PBS 1× twice to remove any residue, and 100 μL of MTT were added (1× = 5 mg/mL dissolved in medium without serum) per well and incubated for 4 h at 37 °C to allow the metabolism and formation of formazan crystals in darkness. At the end of this period, the supernatant of each well was removed and 100 μL of DMSO were added to allow solubilization of the formazan. The assays were performed in three independent replicates by triplicate. With the absorbance values obtained, the percentage of cell viability was calculated with the following formula:

% Viability = (ODtreatment cells/ODcontrol cells) × 100(2)

The IC_50_ was calculated by means of a nonlinear regression analysis (percentage of viability vs. concentration Log) with the GraphPad Prism software (v7.00 GrapPad Software Inc., La Jolla, CA, USA).

### 3.7. Phosphatidylserine Translocation (Annexin V-APC)

We used the Apoptosis Detection Kit (Annexin V-APC Apoptosis Detection Kit BD Pharmigen), and the procedure was performed according to the manufacturer’s protocol. SK-LU-1 cells were seeded in RPMI-1640 medium supplemented with 10% FBS and 1% antibiotic-antimycotic solution. When obtaining sufficient confluency of cells, these were seeded in 6-well plates at a density of 5000 cells/well, they were left 24 h to allow the adherence of the cells. After that, the cells were stimulated with the IC_50_ of the extract fraction of *A. lechuguilla*, DMSO (0.1%) was used as a negative control and Paclitaxel (5.0 μg/mL) as positive control. The assays were carried out at 6, 12 and 24 h, after that time the cells were trypsinized and collected in cytometry tubes, centrifuged at 1400 RPM for 5 min, and the supernatant was discarded.

The cells were resuspended in 500 μL of binding buffer (1×), 1 μL of Annexin V-APC and 1 μL of 7-AAD were added and the samples were incubated for 15 min in the dark. Finally, samples were read on an Accuri flow cytometer (Becton Dickinson, San Diego, CA, USA), and analyzed with the FlowJo software (v10.4.2 LLC, Ahsland, OR, USA).

### 3.8. In Silico Analysis

To explore the structural mechanism by which these molecules present in the fractionated hydrolyzed extract of *lechuguilla*, an *in silico* analysis through molecular docking was performed. Computational dockings calculations were carried out by using the DockingServer [53]. Employing Fas/CD95 [2NA7], TNF-R1 [2ZJC], DR4 [5CIR], DR5 [1D0G], tBID [2M5I], Bax [2G5B], Bak [2JCN], BCl-2 [5FCG], Casp-3 [1NME], Casp-8 [3KJQ], RIP1 [4ITJ], FADD [1E3Y], MLKL [4M67], ALK [2YHV], EGFR [1IVO], K-Ras [3GFT], TGFBR [3KED], MEK [3MBL], and MAPK [3TG3] the codes in square bracket refer to protein data bank entries (protein crystal structures). Molecules were downloaded from PubChem and the docetaxel and dimer of flavonoid were designed by using ChemProfessional 15.0 (v15.0.0.106 Chemdraw, PerkinElmer Informatics, MA, USA). Gaesteiger partial charges were added to the ligand atoms. Non-polar hydrogens atoms were merged, and rotatable bonds were defined. The MMFF94 force field was used for the energy minimization of ligand molecules. Docking simulations were performed by using the LGA. Each docking experiment was derived from 100 different runs that were set to terminate after a maximum of 2,500,000 energy evaluations. The population size was set up to 150. During the search, a translational step of 0.2 Å, and quaternion and torsion steps of 5 were applied.

## 4. Conclusions

The biflavonoid found by mass spectrometry, together with the flavonoids kaempferol and quercetin, turned out to be a mixture with synergistic activity on the cell viability of SK-LU-1, considering the IC_50_, the induction of apoptosis and the possible activation of the signaling pathways both extrinsic and intrinsic, and blocking signaling on the EGFR and K-Ras, were studied through molecular docking. The reduction of cell viability is not related to the antioxidant capacity, it rather depends on the specific interaction of the ligands with the membrane receptors of the signaling pathways of the induction of apoptosis. Molecular docking enabled us to demonstrate that the variety of molecules allows the generation of interactions with death receptors that provoke apoptosis by the extrinsic pathway; as well as through the intrinsic pathway by tBID, as generator of the cytochrome C formation, and consequently, the activation of caspase-3, amplifying the apoptotic signal, in addition to showing possible inhibition in EGFR and K-Ras by stopping the growth of lung cancer cells. In general, the mixture of the three flavonoids (kaempferol, quercetin y biflavonoid) can be used as therapeutic targets, due to the activation of death receptors and caspases-3 and -8.

It is necessary to carry out studies on the interaction of the ligands, receptors and proteins involved in the signaling pathways, along with to evaluating the response of the extract on carcinogenic lines that present the K-Ras mutation as: colon, pancreas or thyroid, as well as the effect of the extract enriched in flavonoids in vivo.

## Figures and Tables

**Table 1:** Concentrations of total polyphenols, total flavonoids and antioxidant capacity.

Extract of *A. lechuguilla*	Total Polyphenols ^1^	Total Flavonoids ^2^	TEAC ^3^	ORAC ^3^
Ethanolic	23.44 ± 1.47	19.62 ± 1.23	87.36 ± 3.57	1962.99 ± 211.86
Hydrolyzed	37.45 ± 3.57	3.08 ± 0.00	46.53 ± 0.72	1216.83 ± 4.88
Fraction	2.69 ± 0.12	1.47 ± 0.00	6.12 ± 0.91	49.20 ± 0.80

^1^ µg Eq gallic acid mg extract^−1^, ^2^ µg Eq quercetin mg extract^−1^, ^3^ µM Eq Trolox mg extract^−1^. Mean values ± SD of replicate samples analyzed in triplicate.

**Table 2:** Ion assignation and formula condensed of metabolities in extracts from *A. lechuguilla*.

Possible Molecule	Rt ^1^	*m*/*z*	Ions Assignation	CF ^2^
**Ethanolic extract of *A. lechuguilla***				
Tigogenin-glycoside or Neotigogenin-glycoside or Smilagenin-glycoside or Sarsasapogenin-glycoside	0.42	417.339 579.396 741.450 903.506 1035.549 1197.605	[M + H] [M + Hex + H] [M + Hex + Hex + H] [M + Hex + Hex + Hex + H] [M + Hex + Hex + Hex + Pent + H] [M + Hex + Hex + Hex + Pent + Hex + H]	C_56_H_92_O_27_
Hecogenin-glycoside or Sisalagenin-glycoside or Gloriogenin-glycoside or Yuccagenin-glycoside	0.56	431.336 593.375 755.429 917.484 1049.528 1211.584	[M + H] [M + Hex +H] [M + Hex + Hex + H] [M + Hex + Hex + Hex + H] [M + Hex + Hex + Hex + Pent + H] [M + Hex + Hex + Hex + Pent + Hex + H]	C_56_H_90_O_28_
Kaempferol	5.06	245.082 269.085 287.096	[M − C_2_H_2_O + H] [M − H_2_O + H] [M + H]	C_15_H_10_O_6_
Quercetin	5.44	257.082 285.080 303.091	[M − H_2_O − CO +H] [M − H_2_O + H] [M + H]	C_15_H_10_O_7_
Diosgenin-glycoside or Yamogenin-glycoside	6.09	415.325 577.381 709.424 871.479 1003.523	[M + H] [M + Hex + H] [M + Hex + Pent + H] [M + Hex + Pent + Hex + H] [M + Hex + Pent + Hex + Hex + H]	C_50_H_80_O_22_
Tigogenin-glycoside or Neotigogenin-glycoside or Smilagenin-glycoside or Sarsasapogenin-glycoside	6.34	417.342 579.397 741.451 903.506 1065.561 1197.606	[M + H] [M + Hex + H] [M + Hex + Hex + H] [M + Hex + Hex + Hex + H] [M + Hex + Hex + Hex + Hex + H] [M + Hex + Hex + Hex + Hex + Pent + H]	C_56_H_92_O_27_
Tigogenin-glycoside or Neotigogenin-glycoside or Smilagenin-glycoside or Sarsasapogenin-glycoside	6.57	417.341 579.396 741.451 903.506 1035.550	[M + H] [M + Hex + H] [M + Hex + Hex + H] [M + Hex + Hex + Hex + H] [M + Hex + Hex + Hex + Pent + H]	C_50_H_82_O_22_
**Hydrolyzed extract of *A. lechuguilla***				
Unknow	0.54	701.503 475.332 340.264	ND	ND
Unknow	0.68	701.503 475.332	ND	ND
Kaempferol	4.97	245.082 269.085 287.096	[M − C_2_H_2_O + H] [M − H_2_O + H] [M + H]	C_15_H_10_O_6_
Quercetin	5.34	245.082 257.085 261.135 285.080 303.091	[M − C_2_H_2_O_2_ + H] [M − H_2_O − CO + H] [M − C_2_H_2_O + H][M − H_2_O + H] [M + H]	C_15_H_10_O_7_
Diosgenin or Yamogenin	5.36	415.155	[M + H] [M + Na + H]	C_27_H_42_O_3_
Tigogenin or Neotigogenin or Smilagenin or Sarsasapogenin	6.92	417.342	[M + H]	C_27_H_44_O_3_
Unknow	7.12	449.326	ND	ND
Biflavonoid (isomer)	8.37	230.251 245.083 261.135 304.304 579.397	[Quercetin − 2CO − H_2_O + H] [Quercetin − C_2_H_2_O_2_ + H] [Quercetin − C_2_H_2_O + H] [Quercetin + 2H] [Quercetin + Afzelequin + 3H]	C_30_H_22_O_12_
Biflavonoid (isomer)	8.56	230.251 245.083 261.135 304.304 579.397	[Quercetin − 2CO − H_2_O + H] [Quercetin − C_2_H_2_O_2_ + H] [Quercetin − C_2_H_2_O + H] [Quercetin + 2H] [Quercetin + Afzelequin + 3H]	C_30_H_22_O_12_
Hecogenin or Sisalagenin or Gloriogenin or Yuccagenin.	10.01	431.296 453.216	[M + H] [M + Na + H]	C_27_H_42_O_4_
**Extract fraction of *A. lechuguilla***				
Kaempferol	4.99	245.082 269.085 287.096	[M − C_2_H_2_O + H] [M − H_2_O + H] [M + H]	C_15_H_10_O_6_
Quercetin	5.33	257.085 285.080 303.091	[M − H_2_O − CO + H] [M − H_2_O + H] [M + H]	C_15_H_10_O_7_
Unknow	6.48	269.086 365.111	ND	ND
Biflavonoid (isomer)	8.54	245.083 304.306 579.399 601.381	[Quercetin − C_2_H_2_O_2_ + H] [Quercetin + 2H] [Quercetin + Afzelequin + 3H] [Quercetin + Afzelequin + Na + 3H]	C_30_H_22_O_12_
Biflavonoid (isomer)	8.88	245.083 304.306 579.399 601.381	[Quercetin − C_2_H_2_O_2_ + H] [Quercetin + 2H] [Quercetin + Afzelequin + 3H] [Quercetin + Afzelequin + Na + 3H]	C_30_H_22_O_12_

^1^ Retention time in minutes; ^2^ CF, condensed formula; Afzelequin: 274 *m*/*z*; Hex: C_6_H_10_O_5_ Hexose 162 *m*/*z*; Pent: C_5_H_8_O_4_ Pentose 132 *m*/*z*; ND, Not determined. Supplementary materials (Figure S1–S22) show the interpretation of the mass spectra.

**Table 3:** Percentage of cellular inhibition of ethanolic extract of *A. lechuguilla*.

Grow Inhibition ^1^ [%]						
Control	HCT-15	MCF-7	PC-3	U-251	SK-LU-1	K-562
1.2 ± 0.5	33.4 ± 3.6	10.5 ± 4.0	11.5 ± 1.8	24.0 ± 2.6	75.7 ± 2.3	17.1 ± 1.0

^1^ Concentration of treatment of 50 µg *A. lechuguilla* extract/mL; Control, Cos-7 monkey kidney cells; Mean values ± SD of replicate samples analyzed in duplicate.

**Table 4:** Cells of SK-LU-1 response to treatment with fraction extract of *A. lechuguilla*.

Treatment [h]	Live Cells	Early Apoptotic Cells	Late Apoptotic Cells	Necrosis Cells	Total Apoptosis
	[%]				
Control	87.00	3.19	6.72	3.13	9.91
6	79.50	3.56	11.00	5.96	14.56
12	66.20	8.11	12.00	13.70	20.11
24	59.30	17.30	22.5	0.99	39.80

**Table 5:** Summary of free energy, inhibition constant (*K*_i_) and molecular interactions from metabolites determinate in fraction extract of *A. lechuguilla*.

Molecular Docking			Hydrogen Bonds		Polar Interactions		Hydrophobic Interactions	
Protein/Enzyme	Free Energy ^a^/*K*_i_^b^	Ligand	Atom/aa	DST ^c^	Atom/aa	DST ^c^	Atom/aa	DST ^c^
**Death Receptor Signaling**								
Fas/CD95	+0.38/--- +0.13/--- +0.13/--- +0.48/---	Docetaxel Kaempferol Quercetin Biflavonoid	--- O1/W189 --- ---	--- 3.18 --- ---	--- --- --- ---	--- --- --- ---	--- --- --- ---	--- --- --- ---
TNF-R1	−6.35/245.01 +21.13/--- −5.03/206.98 −4.63/405.84	Docetaxel Kaempferol Quercetin Biflavonoid	O8/T89 O11/T89 --- --- --- O6/T89 O7/T89 O5/T147 --- --- --- O3/T89 --- --- --- --- --- --- --- ---	3.11 3.44 --- --- --- 2.89 3.01 3.09 --- --- --- 2.81 --- --- --- --- --- --- --- ---	O12/T89 H4/T89 O2/T89 O9/T89 --- H3/T89 H4/T89 O4/N92 O3/N92 H2/N34 H2/T147 O9/S81 H18/S81 H14/T89 O9/N92 H18/N92 H17/E135 O7/E135 H16/E135 O8/E135	3.71 3.35 3.84 3.50 --- 2.23 2.05 3.70 3.81 3.76 3.63 3.37 3.49 2.04 3.00 2.07 1.99 3.03 2.13 2.90	--- --- --- --- --- C14/P90 C15/P90 C8/V91 --- --- --- C13/P90 C30/P90 C21/P90 C24/P90 --- --- --- --- ---	--- --- --- --- --- 3.34 3.52 3.48 --- --- --- 3.75 3.64 3.61 3.80 --- --- --- --- ---
DR4	−4.29/712.86 −4.39/607.34 −4.78/315.05 −5.12/176.35	Docetaxel Kaempferol Quercetin Biflavonoid	O6/R158 --- --- --- --- --- --- --- --- --- --- --- --- --- --- O1/T129 O5/T129 --- --- --- ---	3.33 --- --- --- --- --- --- --- --- --- --- --- --- --- --- 3.41 3.02 --- --- --- ---	H4/T129 O7/E155 H3/E155 O7/R158 H3/R158 O5/R158 H2/R158 H2/T149 H15/T129 O1/T129 O1/E155 O2/E155 O4/E155 O2/R158 O5/H270 O1/E155 O2/E155 O4/E155 O2/R158 H15/T129 O5/H270 --- --- ---	3.51 2.90 2.00 3.49 3.41 3.80 3.68 3.67 2.26 3.75 3.36 3.31 3.86 3.31 3.38 3.87 3.64 3.46 3.17 2.26 3.38 --- --- ---	--- --- --- --- --- --- --- --- --- C13/V152 --- --- --- --- --- --- --- --- --- C23/V152 C24/V152 C30/V152 C13/C180 C14/C180	--- --- --- --- --- --- --- --- --- 3.26 --- --- --- --- --- --- --- --- --- 3.35 3.61 3.39 3.72 3.29
DR5	−1.73/53.64 × 10^3^ −5.85/6.76 × 10^3^ +6.28/--- +204.31/---	Docetaxel Kaempferol Quercetin Biflavonoid	O7/S22 O5/S22 H2/S22 H2/P23 --- --- --- --- ---	3.15 3.17 3.50 3.72 --- --- --- --- ---	--- O3/R39 H1/R39 O1/D40 O2/D40 O4/D40 H1/D40 --- ---	--- 3.55 3.16 3.08 3.77 3.64 3.85 --- ---	--- --- --- --- --- --- --- --- ---	--- --- --- --- --- --- --- --- ---
**Mitochondrial Control of Apoptosis**								
tBID	−3.24/4.21 × 10^3^ −2.40/17.55 × 10^3^ −2.69/10.58 × 10^3^ −2.77/9.29 × 10^3^	Docetaxel Kaempferol Quercetin Biflavonoid	O10/R84 O3/S78 O6/R63 O7/R63 O7/R71 O6/R71 O3/S78 O11/R71 O9/S78	2.57 2.19 2.68 1.95 1.76 1.88 1.88 2.95 2.17	--- --- --- --- --- --- --- --- ---	--- --- --- --- --- --- --- --- ---	--- --- --- --- --- --- --- --- ---	--- --- --- --- --- --- --- --- ---
Bax	+0.02/--- −5.33/122.89 +0.13/--- +0.46/---	Docetaxel Kaempferol Quercetin Biflavonoid	--- --- --- --- --- --- --- --- --- --- --- ---	--- --- --- --- --- --- --- --- --- --- --- ---	--- H1/D31 O2/D31 O4/D31 O5/N31 O5/S98 H2/S98 O2/T151 O4/S154 O4/L203 --- ---	--- 3.33 3.36 3.29 3.70 3.52 3.65 3.58 3.57 3.76 --- ---	--- --- --- --- --- --- --- --- --- --- --- ---	--- --- --- --- --- --- --- --- --- --- --- ---
Bak	+0.14/--- +0.51/--- +0.13/--- +0.42/---	Docetaxel Kaempferol Quercetin Biflavonoid	--- --- --- ---	--- --- --- ---	--- --- --- ---	--- --- --- ---	--- --- --- ---	--- --- --- ---
Bcl-2	+0.04/--- +0.51/--- +0.13/--- +0.42/---	Docetaxel Kaempferol Quercetin Biflavonoid	--- --- --- ---	--- --- --- ---	--- --- --- ---	--- --- --- ---	--- --- --- ---	--- --- --- ---
**Caspases**								
Casp-3	−6.79/10.47 −6.15/30.95 +0.13/--- +0.43/---	Docetaxel Kaempferol Quercetin Biflavonoid	O6/Y204 O11/Y204 --- --- --- --- --- --- --- --- O1/H121 O5/Q161 O1/C163 H2/R64 H2/Q161 H2/A162 --- ---	3.40 3.17 --- --- --- --- --- --- --- --- 3.12 3.09 3.44 3.80 3.73 3.57 --- ---	O4/T62 H1/T62 O12/H121 N1/H121 H5/H121 O8/Y204 O2/Y204 --- --- --- O5/R64 O6/E123 H3/E123 O4/Y204 H1/Y204 O3/R207 --- ---	3.83 3.52 3.53 3.71 2.90 3.07 3.57 --- --- --- 3.38 2.76 1.82 3.26 3.59 3.62 --- ---	C26/H121 C43/C163 C35/C163 C29/C163 C33/C163 C38/C163 C8/F256 C4/F256 C23/F256 C21/F256 C1/C163 C2/C163 C3/C163 C4/C163 --- --- --- ---	3.71 3.17 3.25 3.83 3.41 3.75 3.76 3.76 3.25 3.58 3.45 3.35 3.76 3.66 --- --- --- ---
Casp-8	−1.94/38.07 × 10^3^ −1.05/170.68 × 10^3^ −1.13/147.65 × 10^3^ −0.41/502.84	Docetaxel Kaempferol Quercetin Biflavonoid	--- --- --- --- --- --- --- --- ---	--- --- --- --- --- --- --- --- ---	--- O1/L224 --- --- --- --- --- --- ---	--- 3.20 --- --- --- --- --- --- ---	C39/K224 --- C7/K224 C11/K224 O3/K224 C1/K224 C6/L224 C27/K224 O8/K224	3.55 --- 3.68 3.56 3.75 3.67 3.75 3.53 3.88
**Necroptosis**								
RIP1	ND −7.66/2.44 −7.64/2.53 +63.84/---	Docetaxel Kaempferol Quercetin Biflavonoid	ND --- --- --- --- --- --- --- --- --- --- --- --- --- --- --- --- --- --- --- ---	ND --- --- --- --- --- --- --- --- --- --- --- --- --- --- --- --- --- --- --- ---	ND O5/N68 H2/R71 --- --- --- --- --- --- --- --- O5/H136 H2/H136 --- --- --- --- --- --- --- ---	ND 3.68 3.65 --- --- --- --- --- --- --- --- 3.72 2.91 --- --- --- --- --- --- --- ---	ND C10/L70 C8/L78 C11/L78 C13/L78 C13/L90 C15/L90 C11/M92 C13/M92 C15/M92 C14/M92 C7/V75 C11/V75 C2/V76 C3/V76 C14/L78 C14/M92 C12M92 C15/M92 C11/L129 ---	ND 3.42 3.38 3.34 3.62 3.41 3.65 3.48 3.03 3.11 3.32 3.66 3.55 3.66 3.59 3.89 3.41 3.87 3.88 3.44 ---
MLKL	ND −4.98/225.37 −5.28/135.30 +0.58/---	Docetaxel Kaempferol Quercetin Biflavonoid	ND --- --- --- --- --- --- --- --- --- --- O1/L230 --- --- --- --- --- --- --- --- --- ---	ND --- --- --- --- --- --- --- --- --- --- 3.00 --- --- --- --- --- --- --- --- --- ---	ND O3/R210 H1/R210 O1/K230 H2/K230 O5/E250 H2/E250 O2/S335 O1/N336 O2/N336 H3/E351 O3/R210 O7/Q236 H4/Q236 O5/E250 H2/E250 O6/K331 H3/L331 O1/N336 O2/N336 O4/N336 ---	ND 3.46 3.85 3.39 3.77 3.06 2.09 3.85 3.59 3.67 2.16 3.81 3.38 3.74 3.00 2.06 3.21 3.30 3.88 3.79 3.42 ---	ND C10/L338 C9/A348 C10/A348 --- --- --- --- --- --- --- C11/L338 C9/A348 C11/A348 --- --- --- --- --- --- --- ---	ND 3.59 3.56 3.65 --- --- --- --- --- --- --- 3.66 3.42 3.46 --- --- --- --- --- --- -- ---
FADD	ND +0.51/--- +0.13/--- +0.45/---	Docetaxel Kaempferol Quercetin Biflavonoid	ND --- --- ---	ND --- --- ---	ND --- --- ---	ND --- --- ---	ND --- --- ---	ND --- --- ---
**Epidermal Growth Factor Receptor (MAPK/ERK Pathway), Transforming Growth Factor B Receptor & Multidrug Resistance Protein 1**								
EGFR	ND −6.16/30.34 −6.82/10.10 +7.77/---	Docetaxel Kaempferol Quercetin Biflavonoid	ND O2/S262 O4/S262 --- --- --- --- O2/S262 O4/S262 --- --- --- --- --- ---	ND 3.20 2.88 --- --- --- --- 2.96 2.82 --- --- --- --- --- ---	ND H2/H280 O6/D238 H3/D238 O3/H280 O4/S282 --- O5/H280 H2/H280 O6/D238 H3/D238 H4/D238 --- --- ---	ND 3.16 3.07 2.27 3.61 3.77 --- 3.87 3.05 2.85 1.89 3.72 --- --- ---	ND C8/P242 C15/L245 C14/L245 C12/L245 C9/P242 C10/P242 C8/P242 C13/L245 C15/L245 C14/L245 C10/L245 C11/P242 C9/P242 ---	ND 3.11 3.51 3.01 3.28 3.40 3.34 3.08 3.10 3.39 3.80 3.51 3.20 3.55 ---
TGFβ receptor	ND +0.51/--- +0.13/--- +0.44/---	Docetaxel Kaempferol Quercetin Biflavonoid	ND --- --- ----	ND --- --- ---	ND --- --- ---	ND --- --- ---	ND --- --- ---	ND --- --- ---
K-Ras	ND −6.72/11.93 −7.45/3.45 −6.59/14.71	Docetaxel Kaempferol Quercetin Biflavonoid	ND --- --- --- --- --- --- --- --- O5/S145 H2/D119 H2/S145 H2/K147 O12/K117 H21/L120 --- --- --- --- ---	ND --- --- --- --- --- --- --- --- 3.43 3.07 3.60 3.81 3.36 3.86 --- --- --- --- ---	ND O6/S17 H3/S17 H1/D30 H3/T35 O5/D119 H2/D119 O5/S145 H2/S145 O7/S17 H4/S17 O5/D119 --- H18/S17 O6/D30 H16/D30 H15/D119 O5/D119 O4/K147 O5/K147	ND 3.41 2.50 3.88 3.89 3.10 2.18 3.66 3.14 3.63 3.47 2.92 --- 3.57 3.46 3.83 2.00 2.76 3.55 3.54	ND C11/A18 --- --- --- --- --- --- --- C4/A18 --- --- --- C9/Y32 --- --- --- --- --- ---	ND 3.32 --- --- --- --- --- --- --- 3.88 --- --- --- 3.81 --- --- --- --- --- ---
ALK	ND −3.01/6.25 × 10^3^ −2.77/9.29 × 10^3^ +180.68/---	Docetaxel Kaempferol Quercetin Biflavonoid	ND --- --- --- --- --- --- --- --- --- --- --- ---	ND --- --- --- --- --- --- --- --- --- --- --- ---	ND O5/E1167 H2/E1167 O6/D1203 H3/D1203 H2/D1270 O5/D1270 O7/N1254 H4/N1254 O6/D1270 H3/D1270 O7/D1270 ---	ND 3.53 3.74 3.01 2.19 2.74 2.71 3.54 3.13 3.13 2.48 3.72 ---	ND C3/L1256 C5/L1256 O6/L1256 C11/L1256 C13/L1256 --- C1/L1256 C2/L1256 C6/L1256 C7/L1256 C8/L1256 ---	ND 3.74 3.37 3.46 3.17 3.44 --- 3.31 3.42 3.53 3.59 3.42 ---
MEK	ND +0.51/--- +0.14/--- +0.90/---	Docetaxel Kaempferol Quercetin Biflavonoid	ND --- --- ---	ND --- --- ---	ND --- ---- ---	ND --- --- ---	ND --- --- ---	ND --- --- ---
MAPK	ND −3.34/3.58 × 10^3^ −3.43/3.08 × 10^3^ −2.64/11.70 × 10^3^	Docetaxel Kaempferol Quercetin Biflavonoid	ND O4/S51 --- --- --- --- --- O7/S51 H4/N49 H4/S51 O12/S51 H21/S51 ---	ND 3.03 --- --- --- --- --- 3.32 3.55 2.93 3.22 3.31 ---	ND O3/N49 H1/N49 O2/S51 O3/S51 H1/S51 H3/R56 O2/S51 H1/R56 O7/N49 O3/N49 H14/N49 ---	ND 3.47 3.57 3.67 3.76 3.88 3.12 3.78 3.13 2.91 3.90 3.54 ---	ND C12/L53 --- --- --- --- --- --- --- --- C22/V35 C23/V35 C19/P33	ND 3.54 --- --- --- --- --- --- --- --- 3.81 3.80 3.89
MRP1	ND +0.50/--- +0.13/--- +0.41/---	Docetaxel Kaempferol Quercetin Biflavonoid	ND --- --- ---	ND --- --- ---	ND --- --- ---	ND --- --- ---	ND --- --- ---	ND --- --- ---

^a^ Kcal/mol; ^b^ µM; ^c^ Distance [Å]; ND: Not determined; ---: No result generated; Å: angstroms; Atom: C—carbon; H—hydrogen, O—oxygen; aa—amino acids; amino acid nomenclature: C—cysteine, H—histidine, I—isoleucine, M—methionine, S—serine, V—valine, A—alanine, G—glycine, L—leucine, P—proline, T—threonine, F—phenylalanine, R—arginine, Y—tyrosine, W—tryptophan, D—aspartic acid, N—asparagine, E—glutamic acid, Q—glutamine, and K—lysine.

## Supplementary Materials

Supplementary materials can be found at http://www.mdpi.com/1422-0067/19/12/3765/s1.

## Abbreviations

7-AAD	7-amino-actinomycin D
ACN	Acetonitrile
ALK	Anaplastic lymphoma kinase
APC	Aloficocianina
BID	BH3 interacting-domain death agonist
Caspases	Cysteine aspartate-specific proteases
CML	Chronic myelogenous leukemia
DISC	Death inducing signaling complex
DMSO	Dimethyl sulfoxide
DR	Death receptor
EDTA	2,2′,2″,2‴-(Ethane-1,2-diyldinitrilo) tetraacetic acid
EGFR	Epidermal growth factor receptor
FADD	Fas-associated death domain protein
FBS	Fetal bovine serum
FITC	Fluorescein
IC	Inhibitory concentration
K-Ras	Kirsten rat sarcoma virus
LGA	Lamarckian genetic algorithm
MAPK	Mitogen activated protein kinase
MEK	MARK/ERK kinase
MLKL	Mixed lineage kinase domain like protein
MTT	3-(4,5-diMethyl-2-Thiazolyl)-2,5-diphenyl-2H-Tetrazolium bromide
NSCLC	Not-small cell lung cancer
OD	Optical density
ORAC	Oxygen radical absorbance capacity
PBS	Phosphate buffered saline
PM	Particulate matter
RIP	Receptor-interacting protein
RPM	Revolutions per minute
SCLC	Small cell lung cancer
SRB	Sulforhodamine B
tBID	Truncated BID
TGFβ receptor	Transforming growth factor beta receptor
TEAC	Trolox equivalent antioxidant capacity
TCA	Trichloroacetic acid
TFA	Trifluoroacetic acid
TNF	Tumor necrosis factor
Tris	Tris(hydroxymethyl)aminomethane
UNAM	Universidad Nacional Autónoma de México
