# Supplementary material for: Apoptosis Induction of Agave lechuguilla Torrey Extract on Human Lung Adenocarcinoma Cells (SK-LU-1)
Source: Int J Mol Sci. 2018 Nov 27;19(12):3765. doi: 10.3390/ijms19123765 (PMC6321503; doi:10.3390/ijms19123765)
Supplement: Supplementary file 1 [file ijms-19-03765-s001.pdf]

Supplementary Materials

The spectra analysis from Table 2, are shown in the Figures S1–S22.

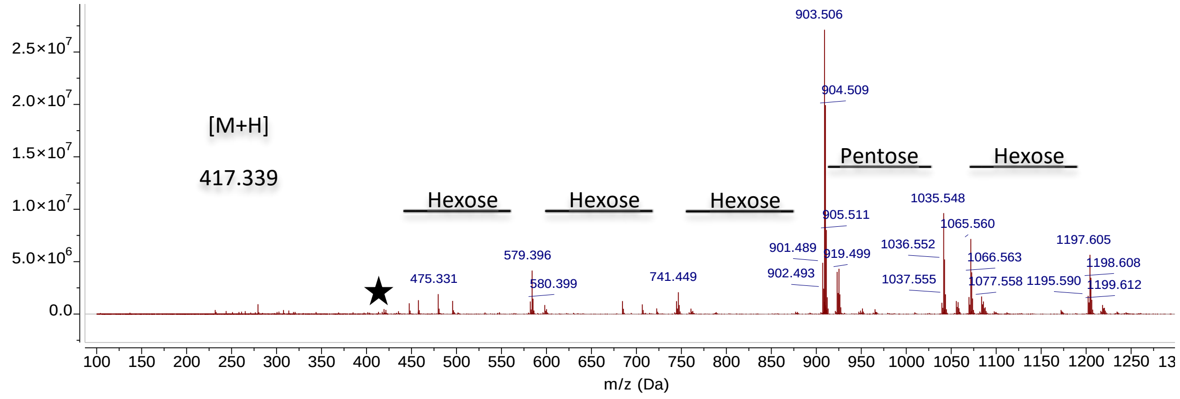

Figure S1. Compound at 0.42 min (Rt) in ethanolic extract of *A. lechuguilla*

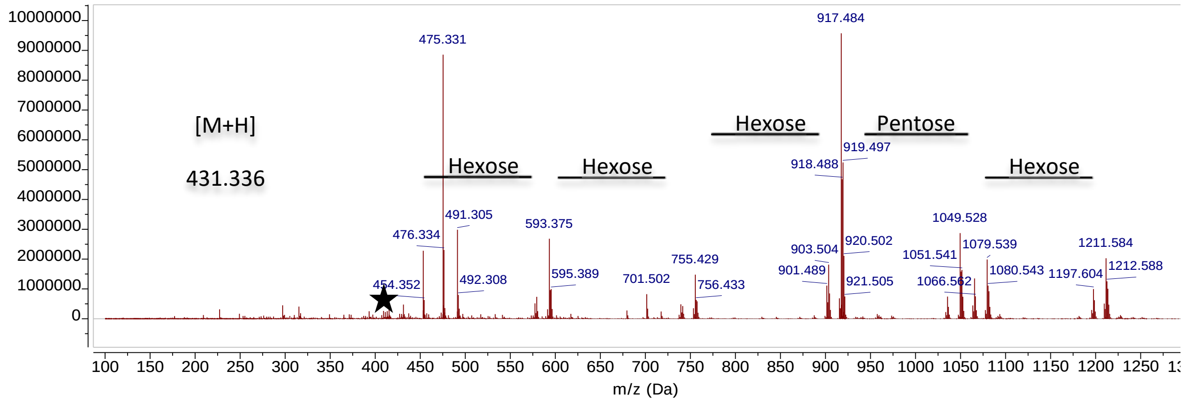

Figure S2. Compound at 0.56 min (Rt) in ethanolic extract of *A. lechuguilla.*

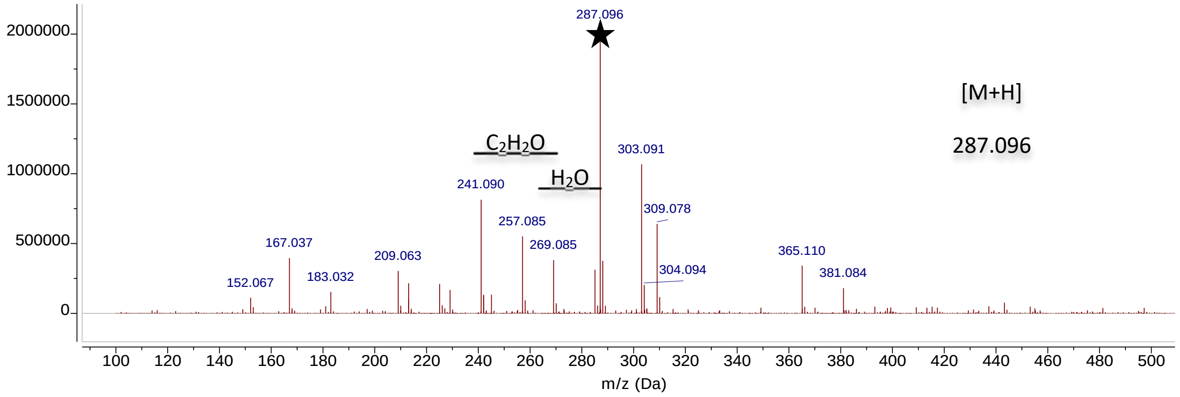

Figure S3. Compound at 5.06 min (Rt) in ethanolic extract of *A. lechuguilla.*

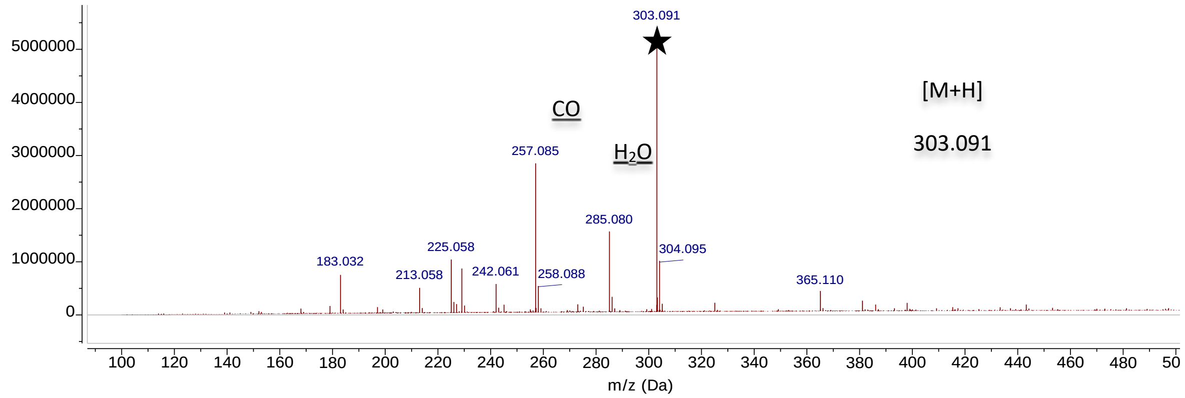

Figure S4. Compound at 5.44 min (Rt) in ethanolic extract of *A. lechuguilla.*

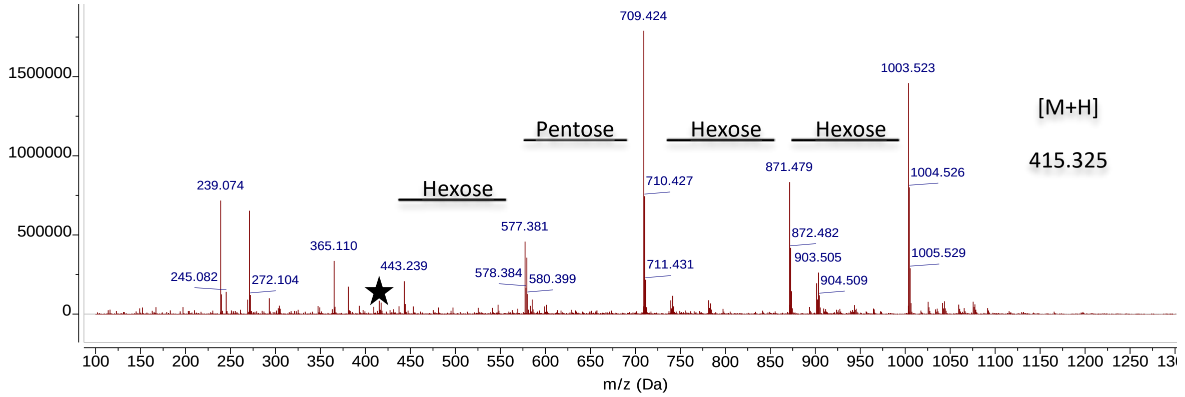

Figure S5. Compound at 6.09 min (Rt) in ethanolic extract of *A. lechuguilla.*

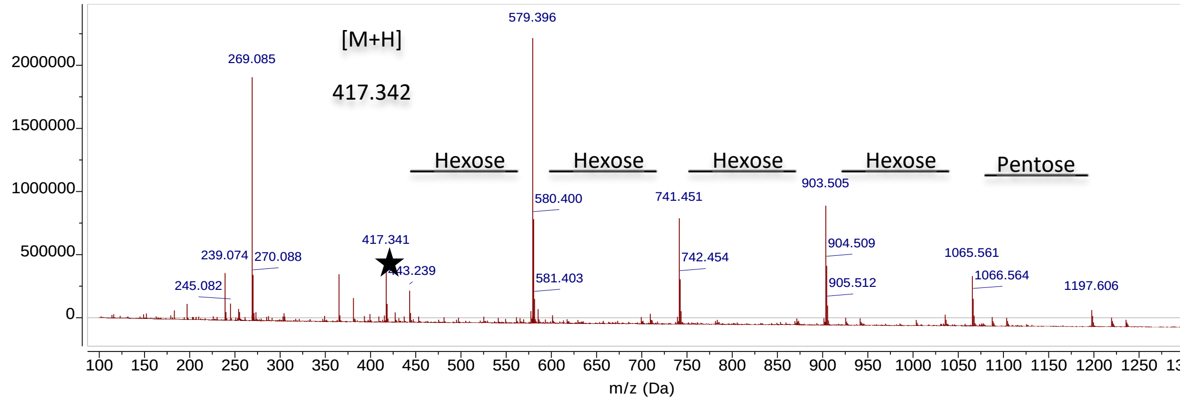

Figure S6. Compound at 6.34 min (Rt) in ethanolic extract of *A. lechuguilla.*

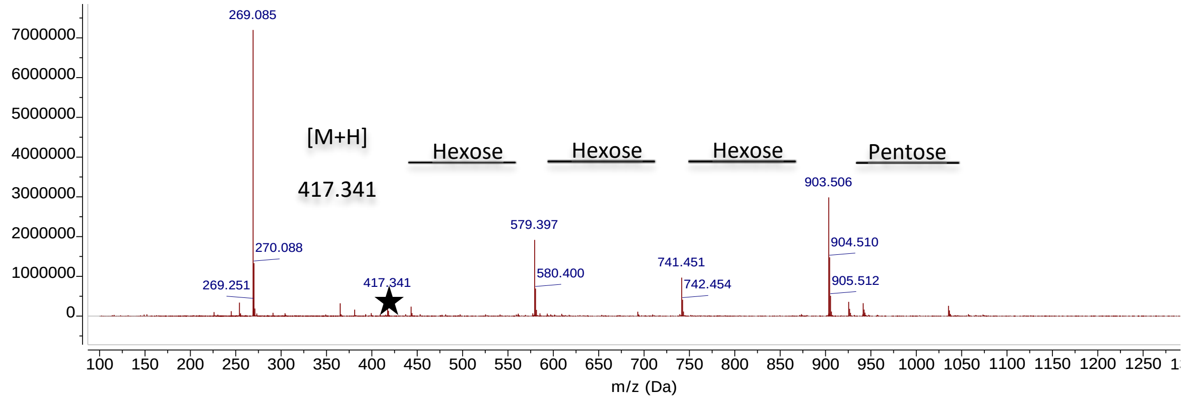

Figure S7. Compound at 6.57 min (Rt) in ethanolic extract of *A. lechuguilla.*

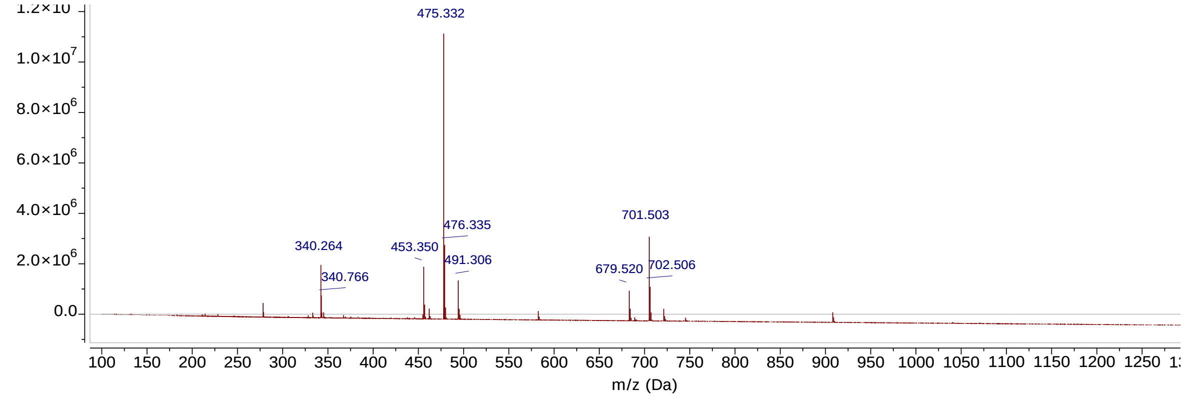

Figure S8. Compound at 0.54 min (Rt) in ethanolic extract of *A. lechuguilla.*

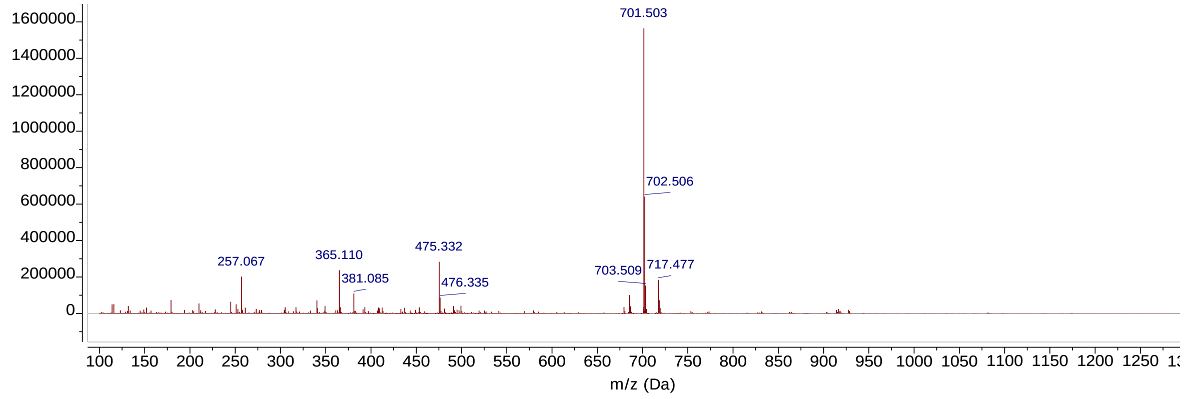

Figure S9. Compound at 0.68 min (Rt) in ethanolic extract of *A. lechuguilla.*

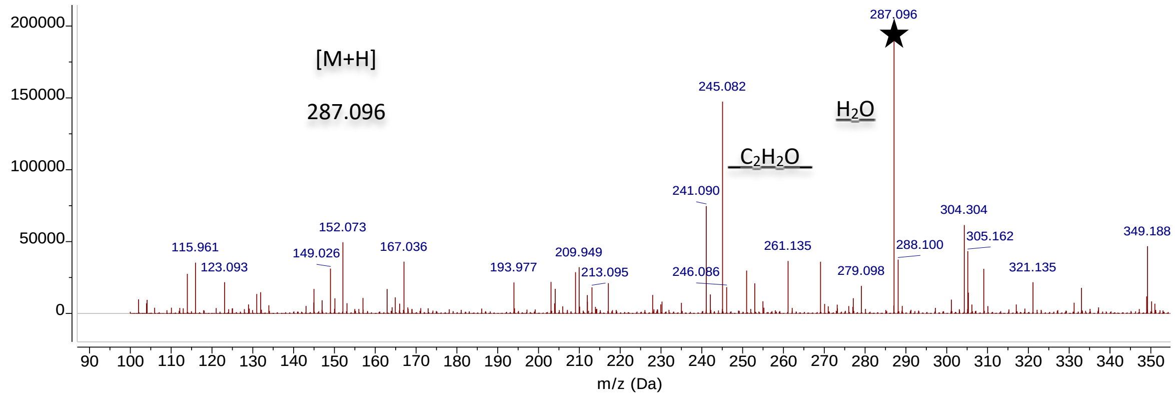

Figure S10. Compound at 4.97 min (Rt) in ethanolic hydrolyzed extract of *A. lechuguilla.*

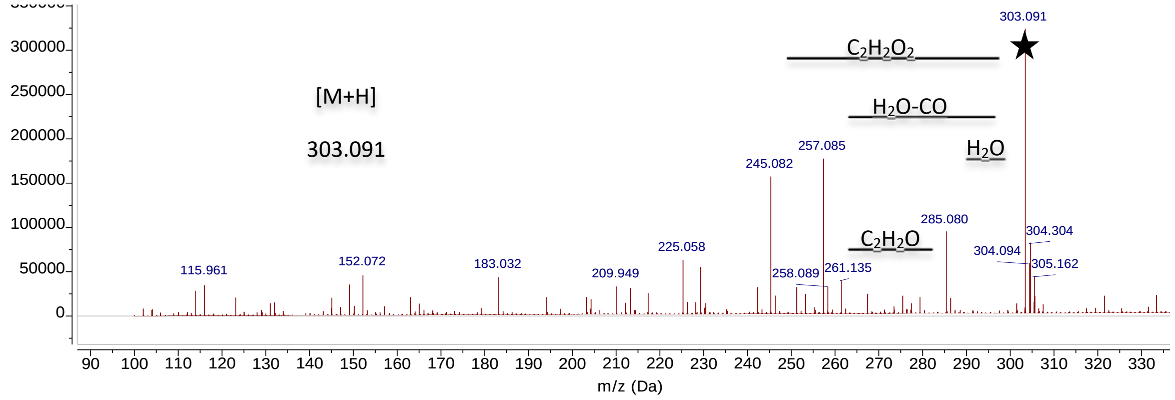

Figure S11. Compound at 5.34 min (Rt) in ethanolic hydrolyzed extract of *A. lechuguilla.*

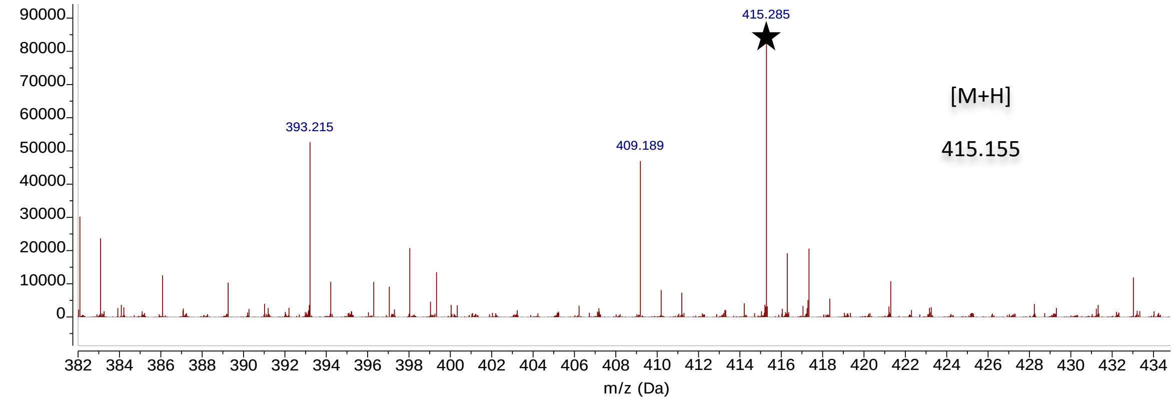

Figure S12. Compound at 5.36 min (Rt) in ethanolic hydrolyzed extract of *A. lechuguilla.*

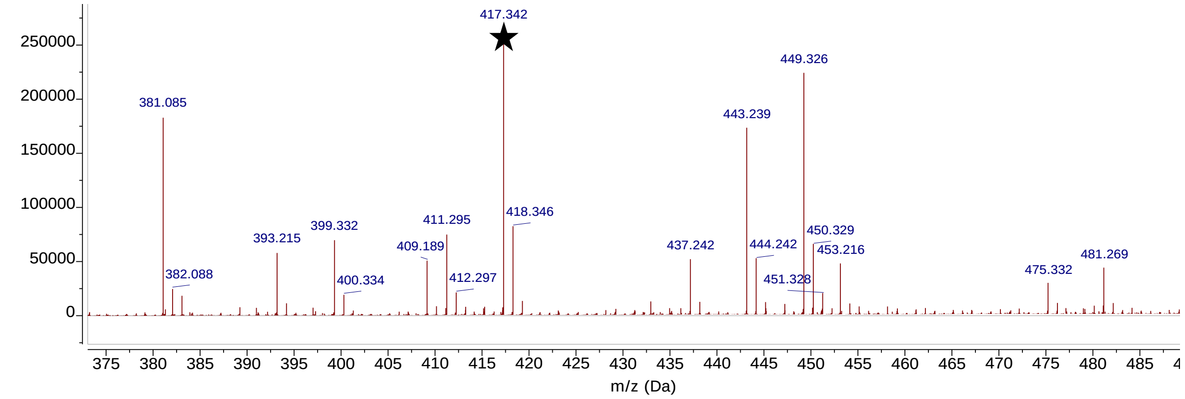

Figure S13. Compound at 6.92 min (Rt) in ethanolic hydrolyzed extract of *A. lechuguilla*.

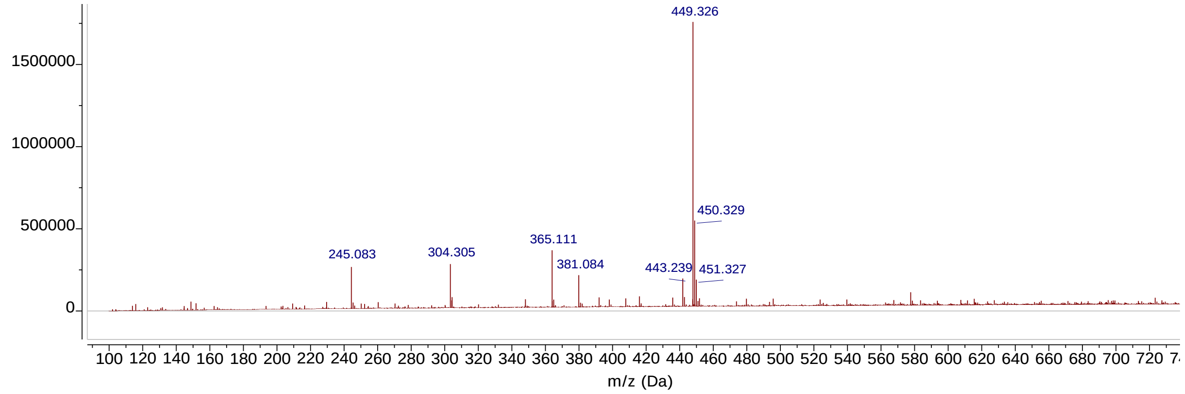

Figure S14. Compound at 7.12 min (Rt) in ethanolic hydrolyzed extract of *A. lechuguilla*.

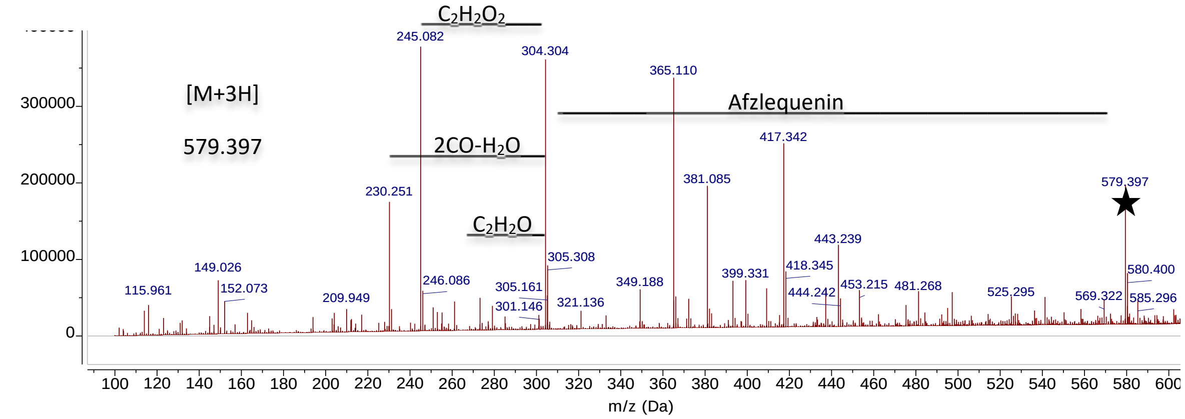

Figure S15. Compound at 8.37 min (Rt) in ethanolic hydrolyzed extract of *A. lechuguilla*.

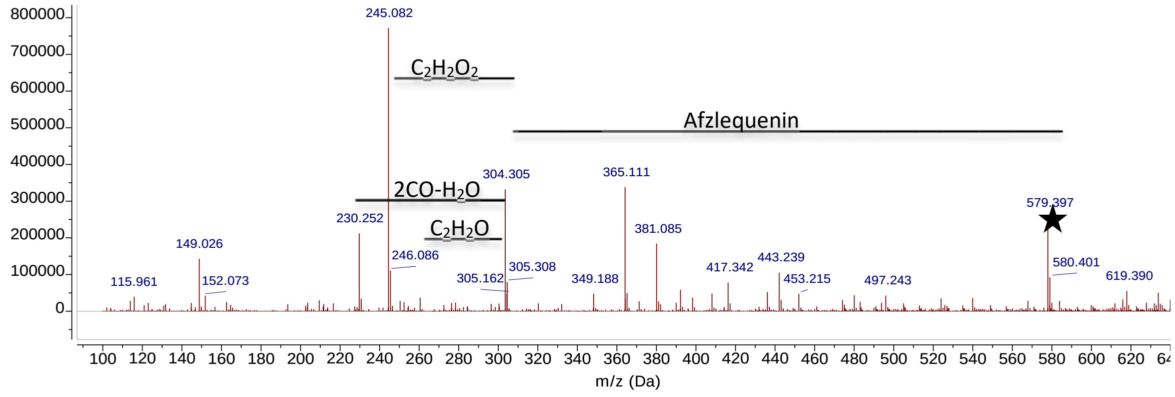

Figure S16. Compound at 8.56 min (Rt) in ethanolic hydrolyzed extract of *A. lechuguilla*.

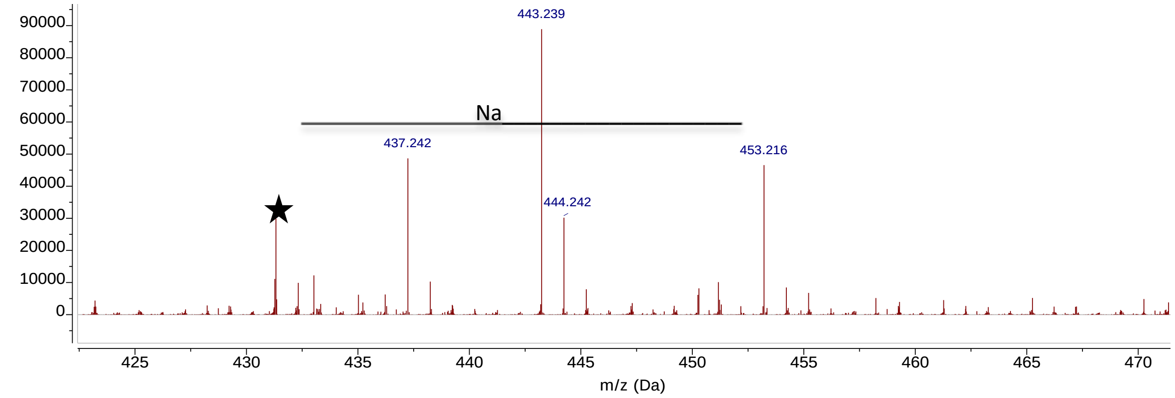

Figure S17. Compound at 10.01 min (Rt) in ethanolic hydrolyzed extract of *A. lechuguilla*.

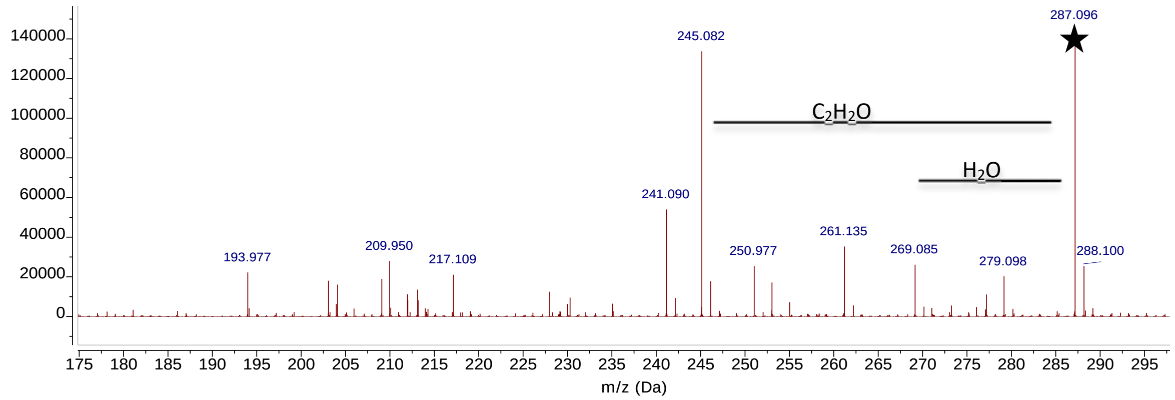

Figure S18. Compound at 4.99 min (Rt) in ethanolic fraction extract of *A. lechuguilla*.

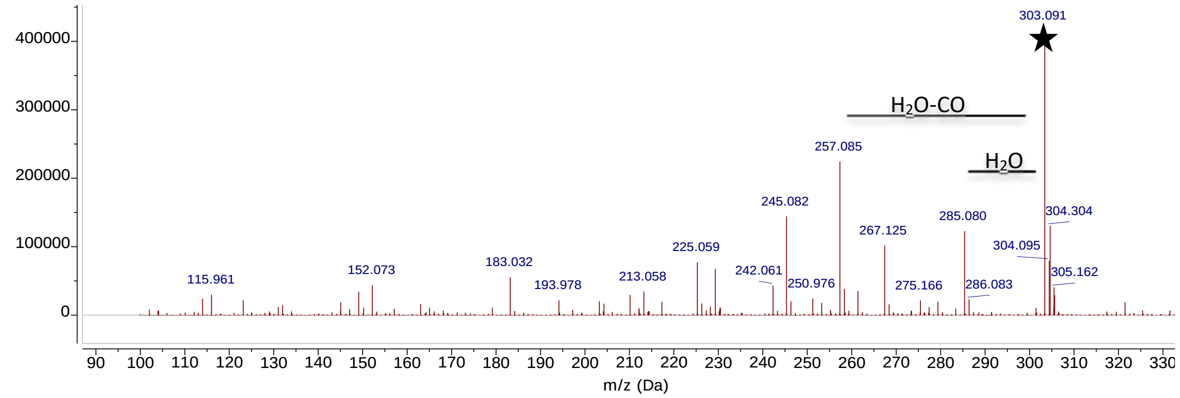

Figure S19. Compound at 5.33 min (Rt) in ethanolic fraction extract of *A. lechuguilla.*

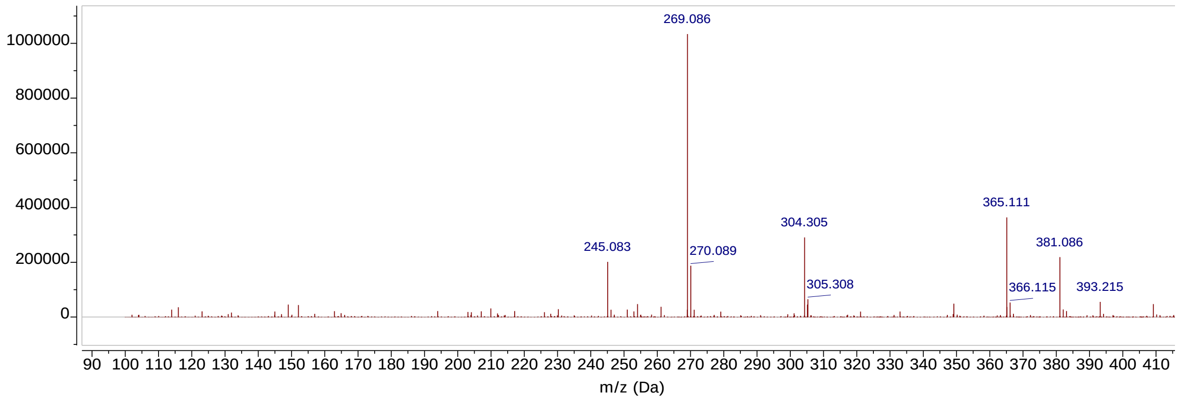

Figure S20. Compound at 6.48 min (Rt) in ethanolic fraction extract of *A. lechuguilla.*

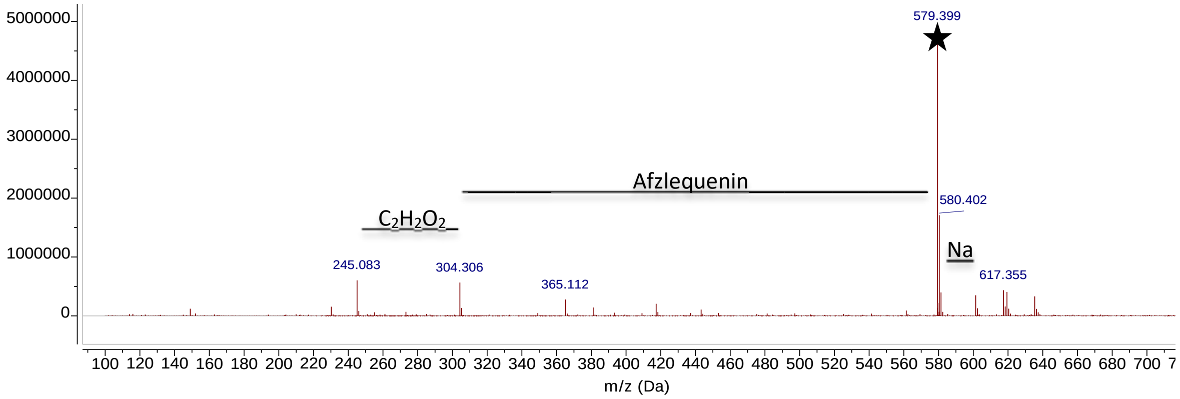

Figure S21. Compound at 8.54 min (Rt) in ethanolic fraction extract of *A. lechuguilla.*

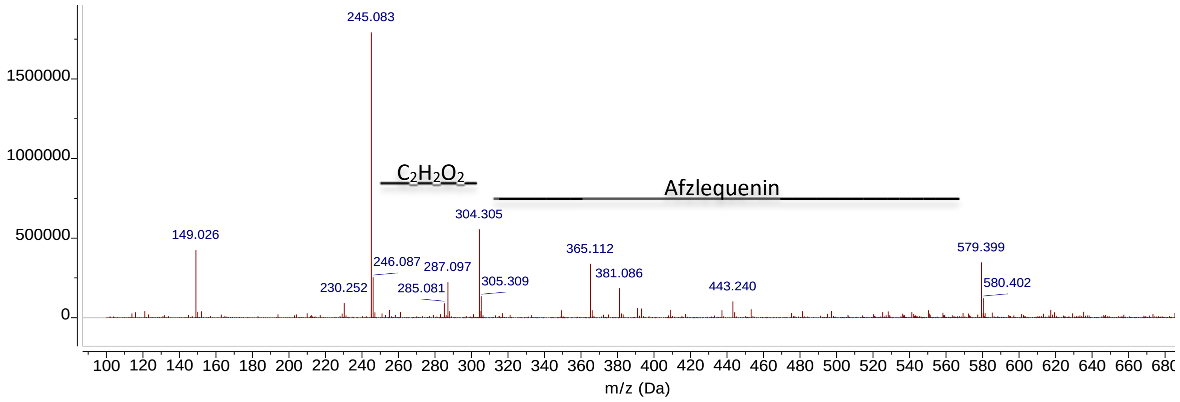

Figure S22. Compound at 8.88 min (Rt) in ethanolic fraction extract of *A. lechuguilla.*
